# *Re-Representing Metaphor:* Modeling Metaphor Perception Using Dynamically Contextual Distributional Semantics

**DOI:** 10.3389/fpsyg.2019.00765

**Published:** 2019-04-15

**Authors:** Stephen McGregor, Kat Agres, Karolina Rataj, Matthew Purver, Geraint Wiggins

**Affiliations:** ^1^LATTICE, CNRS & École Normale Supérieure, PSL, Université Sorbonne Nouvelle Paris 3, Montrouge, France; ^2^Department of Social and Cognitive Computing, Institute of High Performance Computing, A*STAR, Singapore, Singapore; ^3^Department of Psycholinguistic Studies, Faculty of English, Adam Mickiewicz University, Poznań, Poland; ^4^Department of Cognitive Psychology and Ergonomics, University of Twente, Enschede, Netherlands; ^5^Cognitive Science Research Group, School of Electronic Engineering and Computer Science, Queen Mary University of London, London, United Kingdom; ^6^AI Lab, Vrije Universiteit Brussel, Brussels, Belgium

**Keywords:** distributional semantics, metaphor, conceptual models, computational creativity, vector space models, computational linguistics

## Abstract

In this paper, we present a novel context-dependent approach to modeling word meaning, and apply it to the modeling of metaphor. In distributional semantic approaches, words are represented as points in a high dimensional space generated from co-occurrence statistics; the distances between points may then be used to quantifying semantic relationships. Contrary to other approaches which use static, global representations, our approach discovers contextualized representations by dynamically projecting low-dimensional subspaces; in these *ad hoc* spaces, words can be re-represented in an open-ended assortment of geometrical and conceptual configurations as appropriate for particular contexts. We hypothesize that this context-specific re-representation enables a more effective model of the semantics of metaphor than standard static approaches. We test this hypothesis on a dataset of English word dyads rated for degrees of metaphoricity, meaningfulness, and familiarity by human participants. We demonstrate that our model captures these ratings more effectively than a state-of-the-art static model, and does so via the amount of contextualizing work inherent in the re-representational process.

## 1. Introduction

Metaphor is a mode of re-representation: words take on new semantic roles in a particular communicative context, and this phenomenon reflects the way that conceptualisation itself emerges during a cognitive agent's interaction with some situation in a dynamic environment. To describe someone as a *fox* will evoke very different properties in a context which emphasizes *cunning* and in one which emphasizes *good looks*. Metaphor, and the attendant transfer of intensional properties from one conceptual domain to another, is therefore not just a matter of semantic encoding; rather, it involves an agent actually perceiving and experiencing the world through a shift in conceptualisation, and correspondingly in cognitive and linguistic representation.

Because metaphor occurs contextually, we hypothesize that the appropriate mode of lexical-semantic representation will have some mechanism for contextual manipulation. With this in mind, we introduce a methodology for constructing *dynamically contextual distributional semantic models*, allowing for the *ad hoc* projection of representations based on the analysis of contextualizing input. This methodology is based on corpus-driven techniques for building lexical semantic representations, and the components of these representations refer to observations about the way that words tend to occur with other words. The ability to analyse these co-occurrence statistics dynamically will give our model the ability to generate representations in the course of a developing, and potentially changing, conceptual context.

While the term *context* is often used in the field of natural language processing to refer explicitly to the textual context in which a word is observed over the course of a corpus, our methodology has been designed to capture something more in line with the sense of context explored by, for instance, Barsalou (1999), who describes the way that a situation in an environment frames the context specific application of a perceptually grounded symbol. Similarly, Carston (2010a) investigates the way that metaphor arises in the course of the production of *ad hoc* concepts in reaction to a particular situation in the world. One of the primary objectives of our methodology is to describe a framework that accommodates a pragmatic stance on conceptual re-representation that is an essential aspect of metaphor.

In practice, we define contexts in terms of *subspaces* of co-occurrence features selected for their salience in relation to a combination of input words. In the experiments described in the following sections, we will seek to classify and rate the metaphoricity of verb-object compositions, using a statistical analysis of the way that each word in the compositional dyad is observed to co-occur with other words over the course of a large-scale textual corpus. So, for instance, if we have a phrase such as “cut pollution,” we will build context-specific representations based on overlaps and disjunctions independently observed in the co-occurrence tendencies of *cut* and *pollution*. These representations are *dynamic* in that they are generated specifically in response to a particular input, and we show how this dynamism can capture the re-representational quality by which metaphor is involved in the production of *ad hoc* concepts.

Importantly, our contextualization methodology is not contingent on discovering actual collocations of the words in a phrase, and in fact it is perfectly conceivable that we should be able to offer a quantitative assessment of the metaphoricity of a particular phrase based on an analysis of a corpus in which the constituent words never actually co-occur in any given sentence. This is because the representation of a word dynamically generated in the context of a composition with another word is contingent on co-occurrence features which are potentially shared between the words being modeled: while the words *cut* and *pollution* could conceivably never have been observed to co-occur in a particular corpus, it is very likely that they will have some other co-occurrences in common, and our methodology uses these secondary alignments to explore contextual re-representations. We predict that it is not only the features of the contextualized word representations themselves, but also the overall features of the subspace into which they are projected (representing a particular conceptual and semantic context), which will be indicative of metaphoricity.

A key element in the development of our methodology for projecting contextualized distributional semantic subspaces is the definition of conceptual salience in terms of an analysis of specific co-occurrence features. These features become the constituents of a geometric mode of metaphoric re-representation, and our hypothesis is that a thorough analysis of the geometry of a contextually projected subspace will facilitate the assessment of metaphoricity in context. The capacity for our model to make on-line selections, as well as its susceptibility to replete geometric analysis, are key strengths that differentiate this from existing quantitative techniques for representing metaphor. Our computational methodology is a variant of an approach developed for context-dependent conceptual modeling (Agres et al., 2015; McGregor et al., 2015); we describe the model and its application to modeling metaphor perception in section 3.

The data that we use here to explore the re-representational capacities of our methodology consists of human ratings of a set of English language verb-object phrases, categorized in equal parts as *literal* non-metaphors, *conventional* metaphors, and *novel* metaphors, with each phrase given a rating by a group of competent English speakers on a one-to-seven Likert scale for *metaphoricity* as well as for *meaningfulness* and *familiarity*. We note that, in the context of this data (described in section 4), metaphoricity has a negative correlation with assessments of both meaningfulness and familiarity. In section 5, we use this data to train a series of regressions geared to learn to predict ratings for different semantic categories based on the statistical geometry of subspaces contextualized by the concept conveyed by a given phrase.

Our methodology lends itself to a thorough analysis of the way different geometric features in a space of weighted co-occurrence statistics indicate metaphoricity. One of our objectives is the extrapolation of features that are particularly salient to shifts in meaning by way of conceptual re-representation, and to this end we develop a methodology for identifying sets of geometric measures that are independently and collectively associated with metaphor.

## 2. Background

We have developed a novel computational model for metaphor processing, designed to treat metaphor as a graded phenomenon unfolding in the context of an agent's interaction with a dynamic environment. In what follows, we seek to ground our own model in research about the way humans process metaphor. This brief survey leads on to a review of what have been some of the leading computational approaches to modeling metaphor. Finally, we review the ways that existing computational approaches do and do not fit into our own theoretical commitments, setting the scene for the presentation of our own model.

### 2.1. Metaphor Processing and Comprehension in Human Participants

Behavioral and electrophysiological research with human participants has gone a long way in clarifying the cognitive mechanisms involved in metaphoric language processing and comprehension. In most behavioral studies, participants decide whether literal and metaphoric sentences make sense (a semantic judgement task), while the reaction times and accuracy are measured and compared across the different sentence types. In electrophysiological studies, in addition to the behavioral data, Event-Related Potentials (ERP) are analyzed. ERPs are brain responses to specific cognitive events, in this case to literal and metaphoric sentences presented to the participants. Both behavioral and ERP studies on metaphor processing have shown that metaphor processing and comprehension are modulated by the conventionality level of metaphoric utterances.

Analyses of behavioral data obtained from participants in response to literal and metaphoric utterances have revealed longer reaction times and lower accuracy rates when participants judge novel metaphors than literal sentences. Conventional metaphoric sentences evoke either shorter reaction times than novel metaphoric, but longer than literal sentences (Lai and Curran, 2013), or comparable reaction times to literal items (Arzouan et al., 2007). In electrophysiological research, two ERP components have garnered particular interest in this line of work. The N400, a negative-going wave elicited between 300 and 500 ms post-stimulus, was first reported in response to semantic anomaly (Kutas and Hillyard, 1984), with meaningless sentences evoking larger N400 amplitudes than meaningful sentences. In line with previous suggestions and a recently proposed single-stream Retrieval-Integration account of language processing, the N400 can be interpreted as reflecting retrieval of information from semantic memory (Kutas and Federmeier, 2000; Brouwer and Hoeks, 2013; Brouwer et al., 2017). Other accounts propose that the N400 can be seen as reflecting both information retrieval and integration (Coulson and Van Petten, 2002; Lai and Curran, 2013). In electrophysiological research on metaphor, novel metaphors evoke larger N400 amplitudes than conventional metaphors, followed by literal utterances, which evoke the smallest N400 amplitudes (Arzouan et al., 2007). This graded effect might reflect an increase in retrieval of semantic information required for complex mappings in the case of metaphoric utterances, which is additionally modulated by the conventionality of the metaphor.

Another ERP component that has recently received attention in the context of metaphor comprehension is the late positive complex (LPC). LPC is a positive-going wave observed between 500 and 800 ms post-stimulus. While LPC amplitudes observed in response to conventional metaphors converge with those for literal utterances, novel metaphors evoke reduced LPC amplitudes (Arzouan et al., 2007; Goldstein et al., 2012; Rataj et al., 2018; Bambini et al., 2019). This reduction is difficult to interpret within the current theories of the LPC, which see this component as reflecting integration of the retrieved semantic information in a given context. Because semantic integration demands are larger for novel metaphoric than literal sentences, as evident in behavioral data, larger LPC amplitudes for novel metaphoric than literal sentences would be expected. Such increases in LPC amplitudes have been reported in studies that used conventional metaphors, or metaphors that were evaluated as neither familiar nor unfamiliar (De Grauwe et al., 2010; Weiland et al., 2014), but not when the tested metaphoric utterances were novel. One possible interpretation of this novel metaphor effect is that because of the difficulty related to establishing novel mappings in the course of novel metaphor processing, access to semantic information that begins in the N400 time window is prolonged and reflected in sustained negativity that overlaps with the LPC, thus reducing its amplitude. Taken together, ERP findings reveal crucial information about the time-course of metaphor processing and comprehension, and point to two cognitive mechanisms, i.e., semantic information retrieval and integration, as the core operations required in understanding metaphoric language.

Several theoretical accounts of metaphor processing and comprehension have been formulated. The *structure mapping model* (Bowdle and Gentner, 2005; Wolff and Gentner, 2011) proposes that understanding metaphoric utterances such as *this classroom is a zoo* require a symmetrical mapping mechanism to align relational commonalities between the source (*zoo*) and target (*classroom*), as well as an asymmetrical mechanism projecting an inference about the source to the target. The *career of metaphor model* (Bowdle and Gentner, 2005) further posits that conventional metaphor comprehension requires a process of categorization, while novel metaphors are understood by means of comparison. Within the conceptual expansion account, the existing concepts are broadened as a results of novel meaning construction (Ward, 1994; Rutter et al., 2012). Conceptual expansion could be seen as creating a re-representation of an existing concept in the process of novel meaning construction. The important questions thus concern the ways the semantic knowledge is retrieved and integrated in the process of metaphoric meaning construction.

### 2.2. Computational Studies

From the perspective of semantic representation, computational approaches to modeling metaphor have typically sought some mechanism for identifying the transference of salient properties from one conceptual domain to another (Shutova, 2015). Some approaches have used structured, logical representations: one early exemplar is the MIDAS system of Martin (1990), which maps metaphors as connections between different conceptual representations, interpreting the semantic import of a metaphor in terms of plausible projections of properties from once concept to another. The system described by Narayanan (1999) likewise builds up conceptual representations as composites of properties, introducing a concept of broader conceptual domains grounded in knowledge about action in the world which can be mapped to one another by identifying isomorphisms in patterns of relationships within each domain. This move opens up a correspondence between computational methodologies and the theory of *conceptual metaphor* outlined by Lakoff and Johnson (1980). Barnden (2008) offers an overview of these and a few other early approaches, tying them in to the rich history of theoretical and philosophical work on metaphor.

Data-driven approaches have often adopted a similar theoretical premise to metaphor (seeking to model cross-domain mappings), but build representations based on observations across large-scale datasets rather than rules or logical structures. So, for instance, the model developed by Kintsch (2000) extracts statistics about dependency relationships between predicates and subjects from a large-scale corpus and then iteratively moves from a metaphoric phrase to a propositional interpretation of this phrase by traversing the relationships implied by these statistics. Similarly, Utsumi (2011) uses co-occurrence statistics to build up representations, pushing labeled word-vectors into a *semantic space* in which geometric relationships can be mapped to predictions about word meaning: proximity between word-vectors in such a space are used to generate plausible interpretations of metaphors. Shutova et al. (2012a) present a comprehensive review of statistical approaches to the computational modeling of metaphor.

A recent development in these approaches (and in natural language processing in general) has been the application *distributional semantic* techniques to capture phrase and sentence level semantics via the geometry of vector spaces. The distributional semantic paradigm has its roots in the theoretical work of Harris (1957), and particularly the premise that words that tend to be observed with similar co-occurrence profiles across large scale corpora are likely to be related in meaning; modern computational approaches capture this by modeling words as vectors in high-dimensional spaces which capture the details of those co-occurrence profiles. Features of these vectors and spaces have been shown to improve performance in natural language processing tasks ranging from word sense disambiguation (Schütze, 1998; Kartsaklis and Sadrzadeh, 2013) and semantic similarity ratings (Hill et al., 2015) to more conceptually structured problems such as analogy completion (Mikolov et al., 2013; Pennington et al., 2014).

A preponderance of computational schemes for traversing corpora and generating mathematically tractable vector-space representations have been developed (see Clark, 2015, for a fairly recent and inclusive survey). However, the basic insight can be captured by imagining a large matrix in which each row is a vector corresponding to a word in our vocabulary. The columns of this matrix—the *co-occurrence dimensions*—correspond to words which have been observed co-occurring with a vocabulary word. The value of the entry at row *w* and column *c* represents the probability of observing vocabulary word *w* in the context of *c*. Words with similar meanings have similar co-occurrence profiles, and thus similar row vectors, and this similarity can now be measured in mathematical terms. Many variants exist: matrix values are often chosen not as raw probabilities but *pointwise mutual information* values (normalizing the raw probabilities for those expected due to the words' overall frequency); matrices are often factorized to reduce dimensionality and smooth the estimates, or learned using neural networks rather than direct statistics (Mikolov et al., 2013). Co-occurrence can be defined at the level of sentence or whole documents, of words or characters, or in terms of syntactic dependency or other semantic relations (Schütze, 1992; Padó and Lapata, 2007; Kiela and Clark, 2014; Levy and Goldberg, 2014a); although it is usually taken as simple lexical co-occurrence within a fixed-width window of words within sentences. Even this simple version can vary in terms of the co-occurrence window width, with some evidence that the slide from small to large co-occurrence windows might correspond to shifts along semantic spectra such as that of concreteness to abstractness (Hill et al., 2013).

In terms of modeling metaphor, distributional semantic models have been used to generate contextually informed paraphrases of metaphors (Shutova et al., 2012b), have played a role as components in more complex classifiers (Tsvetkov et al., 2014), and have even been used to interface between linguistic and visual data (Shutova et al., 2016). The linear algebraic structure of distributional semantic representations lends itself to composition, in that mathematical operations between word-vectors can be mapped to sequences of words, and interpretations of larger linguistic compositions can therefore potentially be pushed into a computational model (Coecke et al., 2011). Gutiérrez et al. (2016) have exploited this aspect of high-dimensional semantic representations to model metaphoric adjective-noun phrases as operations between a vector (representing a noun) and a second-order tensor (representing an adjective), by which the adjective-tensor projects the noun-vector into a new region of a semantic space. So, for instance, *brilliant child* is represented by a composed vector that we might expect to find in the vicinity of words like *intelligent* rather than words like *glowing*.

### 2.3. The Role of Context

These approaches, however, give little attention to the role of *gradedness* and *context* in the processing of metaphor; but many theoretical approaches point out that these play a vital role. The relevance-theoretic *deflationary account* of Sperber and Wilson (2008), for example, proposes that metaphor can be understood as occupying a region within a spectrum (or perhaps more properly, a region in a multi-dimensional landscape) of various linguistic phenomena that come about in the course of communication. Metaphoricity thus exists not as a binary distinction but on a scale, and as part of a larger scale (and we will see this reflected the data described in section 4 below).

Carston (2010b) emphasizes context-specificity: she argues that there are two different modes of metaphor processing, and that what might be thought of as the more basic and on-line mode involves the construction of *ad hoc* concepts. So, to process a metaphoric verb-object phrases such as *murder wonder*, an ephemeral concept of an activity MURDER* has to be formulated on the spot, and in the context of the application of the phrase. Furthermore, the propositional content of the phrase, to the extent we embrace the idea that language is propositional, begins to become blurred as components of imagery and phenomenology begin to infiltrate language. The idea that metaphoric language involves an extemporaneous projection of a new conceptual framework presents a challenge to cognitivist approaches to metaphor, typified by the theory of conceptual metaphors (Lakoff and Johnson, 1980; Gibbs and Tendahl, 2006), in that it requires a capacity for the construction of *ad hoc* spaces of lexical semantic representations susceptible to the influences of a complex and unfolding situation in which communication between cognitive agents is happening.

This approach therefore questions the idea that metaphor involves mappings between established concepts. To take an example from the data we will model below, the conventional metaphor *cut pollution* arguably involves the construction of an *ad hoc* concept cut*, which extends the action denoted by the verb to something that can be done to *pollution*, in line with Carston (2010a). This is in contrast to a cognitive linguistic perspective on metaphor, which would seek to find a sense in which a fixed property of cutting is transferred to the object *pollution*. In the next sections, we show how a computational method can be developed which follows the *ad hoc* concept view, and test its ability to model human judgements.

## 3. Computational Methodology

With a sense of the way that metaphor fits into a broader range of human semantic representations, we now turn to the task of modeling metaphor computationally. Our objective here is to explore whether and how we can apply statistical analysis of large-scale language corpus data to the problem of re-representing metaphor. Working from the theoretical premise that metaphor emerges in a particular semantic context, we use a methodology for systematically generating on-line lexical semantic relationships on the basis of contextualizing information.

### 3.1. Approach

Our approach is based in the standard distributional semantic view of geometric semantic representation: construction of word meanings as vectors or points that are meaningful in terms of their relationship to one another in some appropriate space, defined in terms of word co-occurrence statistics across a large scale corpus. The distinctive feature of our approach, though, is that the semantic re-representation associated with metaphor interpretation will be expressed as projection into a series of geometric subspaces, each determined in an on-line way on the basis of context. Our model, then, like that of Gutiérrez et al. (2016), seeks to represent metaphor in terms of projections in geometric spaces; however, rather than simply use linear algebraic operations to move or compare word representations within a single static space, we propose to model every instance of a metaphoric composition in terms of a newly generated subspace, specific to the conceptual context in which the metaphor occurs.

This subspace is based on a particular composition (in the experiments below, a two-word verb-noun phrase, but the method is general): its dimensions are chosen as the most salient features—the strongest statistical co-occurrence associations—which the words in the phrase have in common. It is thus distinct in its geometry from the space which would be defined for other compositions using one or the other but not both words. We hypothesize that these dimensions will provide us both an appropriate mechanism for specifying *ad hoc* contextualized projections, and adequate measures for modeling the dynamic production of semantic representations; we test this by learning statistical models based on the geometric properties of the subspaces and the relative positioning of the words within them, and evaluating their ability to predict the metaphoricity of the compositional phrases. To be clear, our objective is not to refute the cognitive stance on metaphor; rather, we seek to provide a methodology that accommodates a pragmatic interpretation of metaphor as a means for communication about extemporaneously constructed concepts, an objective that has proved elusive for computational models.

This context-dependent modeling approach was originally developed by Agres et al. (2015), and further developed by McGregor et al. (2015), for the purposes of context-dependent concept discovery. McGregor et al. (2017) showed that a variant could provide a model of the phenomenon of semantic type coercion of the arguments of verbs in sentential context; and Agres et al. (2016) showed that distances in the contextual subspaces were more closely associated with human judgements of metaphoricity than distances in standard static distributional semantic models. Here, our hypothesis is that this can be used to provide a model of metaphor more generally: that the on-line projection of context specific conceptual subspaces can capture the process of re-representation inherent in the construction of the *ad hoc* concepts necessary to resolve the semantics of a non-literal phrase.

### 3.2. Data Cleaning and Matrix Building

In order to select subspaces suitable for the geometric analysis of word-pairs in the context of a set of co-occurrence dimensions, we begin by building a *base space* from co-occurrence statics over a large textual corpus, using standard distributional semantic techniques. We use the English language component of Wikipedia, and begin by applying a data cleaning process which removes punctuation (aside from apostrophes and hyphens), converts all text into lower case, and detects sentence boundaries. The resulting corpus consists of almost 1.9 billion word tokens representing about 9 million word types, spread across just over 87 million sentences.

We consider the 200,000 most frequent word types in the corpus to be our vocabulary, and our base space will accordingly be a matrix consisting of 200,000 rows (vocabulary word types) and some 9 million columns (co-occurrence word types). We use the standard approach of defining co-occurrence simply as observation within a fixed window within a sentence; here we use a symmetric window of 2 × 2 words. While broader windows have been reported as being suited for capturing specific semantic properties, small windows have proved particularly good for modeling general semantic relatedness; as we are seeking to analyse the paradigmatic relationships inherent in distributional semantics, rather than the type of syntagmatic relationships that emerge over a larger number of words, we choose to focus on smaller co-occurrence windows here (Sahlgren, 2008).

For the matrix values we use a variant of pointwise mutual information (PMI): given a vocabulary word *w* and a word *c* observed co-occurring with *w*, a frequency of observed co-occurrences *f*(*w, c*), independent frequencies of *f*(*w*) and *f*(*c*), respectively, and a total count of vocabulary word occurrences *W*, we define the mutual information between *w* and *c* as follows:

$$\begin{array}{r} PMI\left(w,c\right)=\log_{2}\left(\frac{f\left(w,c\right)\times W}{f\left(w\right)\times \left(f\left(c\right)+a\right)}+1\right) \end{array}$$

Here *a* is a smoothing constant applied to weight against the selection of very infrequent dimensions in the contextual projection procedure that will be described below. This value is set to 10,000, based on trial and error, but this value also turns out to be roughly equal to the mean frequency of all co-occurrence words, meaning that the average ratio of frequencies will be approximately halved; PMI values associated with very rare co-occurrence terms will be severely punished, while values for very common co-occurrence terms will be relatively unaffected. The addition of 1 to the ratio of frequencies guarantees that all PMI values will be non-negative, with a value of 0 indicating that the words *w* and *c* never co-occur with one another. It should be noted that this expression is approximately equivalent to the logarithm of the ratio of the joint probability of *w* and *c* co-occurring, skewed by the smoothing constant and the incrementation of the ratio.

This PMI equation is similar to established methods for weighting co-occurrence statistics, but differs in some important ways that are designed to accommodate the contextual and geometric objectives of our own methodology. In a standard statistical approach to distributional semantics, the information theoretical insight of a PMI type measure is that frequent observations of co-occurrences with infrequent words should be given heavily positive weightings. That idea holds for our own approach up to a point, but, as we would like a mechanism for selecting co-occurrence features that are conceptually salient to multiple words, we would like to avoid giving preference to co-occurrence terms that are so infrequent as to be virtually exclusive to a single word or phrase. Adding *a* balances the propensity for distributional semantic models to emphasize extremely unlikely observations, as this factor will have less of an impact on terms that already have a relatively high overall frequency *f*(*c*). By guaranteeing that all our features are non-negative, we can reliably project our word-vectors into contextualized subspaces characterized by not only angular relationships between the word-vectors themselves, but also with a more informative geometry including a sense of extent, center, and periphery. The merits of this approach will be discussed further in section 3.4.

### 3.3. Projecting Contextualized Subspaces

The procedure described in section 3.2 results in a large and highly informative but also sparse matrix of co-occurrence information, where every observed co-occurrence tendency for all the words in our vocabulary is systematically tabulated. To give a sense of the scope of this representational scheme, every one of the 9 million word types that come up in our corpus becomes the label of a co-occurrence dimensions, but the distribution of word frequencies is characterized by the long tail familiar to corpus linguists, with 5.4 million of the 9 million word types in the corpus co-occurring with one of the 200,000 vocabulary words 10 times or less.

Our next task is to establish a set of techniques for extrapolating *ad hoc* representations capturing the contextualization of the semantics associated with a particular denotation, something that is crucial to metaphoric re-representation. The premise we will work off of is the distributional hypothesis, namely, that consistencies in co-occurrence between two lexical semantic representations correspond to semantic relatedness between the words being represented. Building off of this idea, we propose that there should be subsets of co-occurrence dimensions which are salient to particular conceptual contexts. Given the looseness and ambiguity inherent in word use, and the relationship between this and the drift from literal to figurative language, we suggest that there are groups of co-occurrence dimensions that can collectively represent either observed or potential contexts in which a word can take on particular semantic aspects.

Consider the sets of co-occurrence terms with the highest average PMI values for the words *brilliant diamond* and *brilliant child*, the first of which is likely to be interpreted as a literal phrase, the second of which is a metaphor, albeit a conventionalised one:

1. **brilliant diamond** *carat, koh-i-noor, carats, diamonds, diamond, emerald, barbra, necklace, earrings, rose-cut*
2. **brilliant child** *prodigy, precocious, prodigies, molestation, sickly, couple's, destiny's, intellectually, unborn, imaginative*

Here we can see how the alteration in the noun modified by *brilliant* skews the profile of co-occurrence terms with the highest joint mean into two different conceptual spaces. For the literal phrase *brilliant diamond*, we see co-occurrence terms which seem logically associated with denotations and descriptions of gems, such as *emerald* and *carat*, as well as applications such as *earrings* and specifications such as *rose-cut*. In the case of *brilliant child*, on the other hand, we see words which could stand in as interpretations of the metaphor *brilliant*, such as *prodigy*, or, perhaps with some license, *precocious*, as well as terms related generally to children.

In both cases we also note some unexpected terms creeping in. In the case of *brilliant child*, an analysis of the corpus suggests that the inclusion of *destiny's* is a reference to the music group *Destiny's Child*, who are sometimes described by critics cited in our corpus as “brilliant.” A similar analysis of co-occurrences of the name *Barbra* with *brilliant* and *diamond* across Wikipedia reveals that Barbra Streisand has periodically performed with Neil Diamond, and that she is another artist who has often been acclaimed as “brilliant.” These co-occurrences offer up instances of how elements of ambiguity can enter into relationships between distributional semantic representations: while there is always an explanation for the presence of such dimensions in this type of analysis, there is not an interpretation that is particularly coherent conceptually.

One of the strengths of distributional semantic models, though, is that the high-dimensional spaces they inhabit tend to be fairly resilient against noise. This propensity for using dimensionality to support representations that are, overall, semantically apt aligns with our hypothesis that there should be subsets of dimensions which, taken collectively, represent conceptual contexts. We would like to develop a model which allows for the systematic selection of subspaces of co-occurrence dimensions, based on input consisting of individual words, which on the whole capture something of the conceptual context in which these terms might be composed into a phrase. These techniques, we propose, will allow us to project re-representations of the lexical items involved in the phrase that will facilitate the analysis of how their semantics could metaphorically interact.

With this in mind, we propose to explore three different techniques for selecting subspaces based on an analysis of the co-occurrence profiles of two different input words:

1. mean: We take the co-occurrence terms with the highest arithmetic mean PMI value across input words;
2. geom: We take the co-occurrence terms with the highest geometric mean PMI value across input words;
3. indy: We take a concatenation of the co-occurrence terms with the highest PMI values for each word independently.

For the mean technique, given two input words *w*_1_ and *w*_2_, the value for any candidate co-occurrence term *c*_*j*_ is simply:

$$\begin{array}{l} M\left(c\right)=\left(PMI\left(w_{1},c_{j}\right)+PMI\left(w_{2},c_{j}\right)\right)/2 \end{array}$$

We can take the value for every co-occurrence term and then select the top *k* such terms and project our input words into the corresponding space. For the geom technique, we similarly apply the equation for the geometric mean of PMI values:

$$\begin{array}{l} G\left(c_{j}\right)=\sqrt{PMI\left(w_{1},c_{j}\right)\times PMI\left(w_{2},c_{j}\right)} \end{array}$$

Here it should be noted that, while this equation is strictly defined to include PMI values of 0, the outputs for any such terms would be 0, and so we are in practice only interested in co-occurrence terms with non-zero PMI values for both input words. There is not a rational definition for the geometric mean of a set of inputs containing negative numbers, but, returning to Equation (1) above, we recall that our matrix contains only non-negative elements, anyway.

For the indy technique, we apply an additional constraint to avoid selecting a co-occurrence term that has a high PMI value for both input terms twice. We iteratively select the co-occurrence term with the top PMI value for each input, and, if we encounter a term for one input that was already selected for the other input, we move to the next highest scoring term that hasn't already been selected. We carry this process on until we have established a subspace with *k* dimensions.

The final parameter of this component of our model is *k* itself, the dimensionality of the subspaces selected using any of the techniques now defined. For the purpose of experiments reported here, we will use a value of 200. This value is low enough to guarantee that we can define spaces for the geom technique that involve dimensions with non-zero values for both input words, but on the other hand large enough to hopefully build subspaces that are robust against noise and capture some of the conceptual nuance inherent in the interaction between the input terms as a composed phrase. Other values for *k* have been explored elsewhere (McGregor et al., 2015, 2017), and 200 has generally returned good results. In the present work, our objective is to focus on the alignment of our methodology with theoretical stances on semantic re-representation; there is clearly room for further exploration of the model's parameter space in future work.

**Figure 1:**
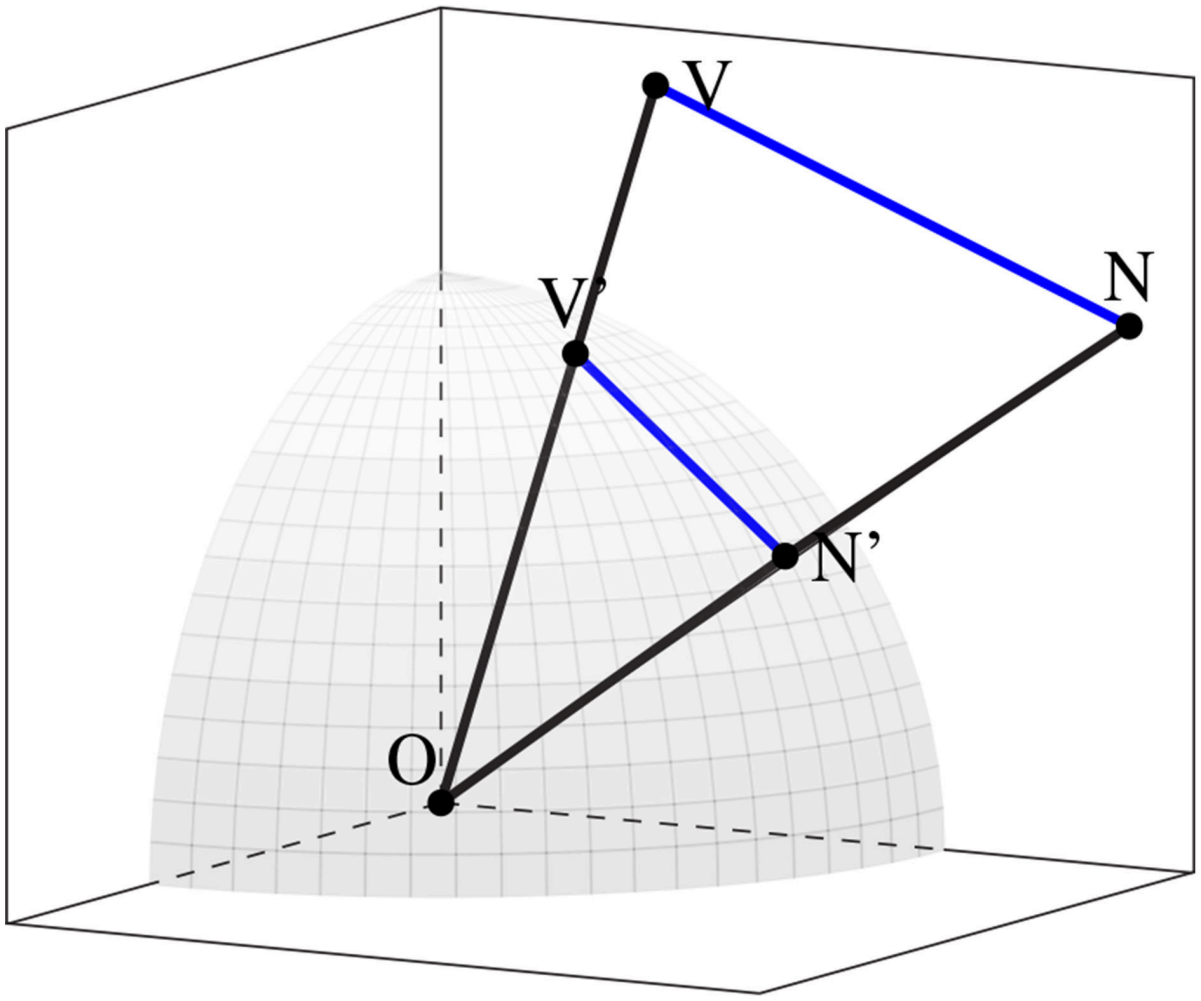
Two word-vectors projected into a contextualized subspace, and the unit sphere intersecting the normalized version of each vector.

An example of a subspace with two word-vectors projected into it is illustrated in Figure 1. Some of the primary element of such a space are also indicated here: in addition to the distance from the origin of each of the word-vectors (represented by the points *V* and *N*), the distance between the vectors $\bar{VN}$ is also an essential measure of the semantic relationship between the two words labeling these vectors, indicating the degree of overlap between these words in the context of the projection they jointly select. Furthermore, a standard technique in distributional semantics is to consider the normalized vectors. To this end, a unit sphere intersecting the vectors is illustrated, and we note that the distance between the normalized vectors *V*′ and *N*′ correlates monotonically with the angle ∠*VON*. These will now serve as a basis for a much more involved analysis of the statistical geometry of a contextualized subspace.

### 3.4. Geometric Analysis of Contextualized Projections

The techniques for analyzing co-occurrence terms associated with potentially metaphoric phrases described in the previous section result in the projection of subspaces in which the word-vectors corresponding to the input words, and for that matter any other word-vector in our base space, maintain a fully geometric aspect. The dimensions of the subspace are labeled by the co-occurrence terms selected, and the values for a word-vector along these dimensions are simply specified by the corresponding value in the full base space.

Because our base space is not normalized, there is, for any word-vector, a notion of distance from the origin of a subspace: the value for any given coordinate of word-vector *w*_*i*_ for co-occurrence dimension *d*_*j*_ will be *PMI*(*w*_*i*_, *d*_*j*_), which could range from 0 if the word never co-occurs with that term to something quite large if the word is on the one hand frequent and on the other hand often co-occurs with a term that is similarly frequent. So, in a given subspace, if a particular word has high PMI values across a number of the co-occurrence dimensions, we would expect it to be far from the origin. Conversely, a word with mainly low and zero PMI values would be close to the origin.

Furthermore, because our subspaces consist only of elements with non-negative values, there is a sense of center and periphery to them. So, for instance, a word-vector with high PMI values for a few co-occurrence dimensions in a given space but low values for most of the dimensions would be skewed away from the center. On the other hand, a word-vector with consistent values across dimensions would be relatively close to the center of the space (though not far from the origin if these values were consistently low).

Word-vectors will naturally have relationships to one another, as well. There is a Euclidean distance between them, an angle between them, and relative distances from the origin. There will also be a number of what we will term *generic vectors* in the space, meaning points corresponding to values characteristic of the space overall rather than any particular word-vector projected into that space. In particular, we define a *mean-vector*, where each element of the vector is the mean value of all word-vectors with non-zero values for each corresponding co-occurrence dimension, a *maximum-vector*, where each element is the highest value for any word-vector along each corresponding dimension, and a *central-vector*, which is simply a uniform vector in which each element is the mean of the mean-vector.

We suggest that these geometric features provide a basis for an analysis of the way in which co-occurrence observations across a large-scale corpus can map to information about metaphoricity and attendant re-representation. In addition to properties such as centrality within the space and distance from the origin discussed above, the relationship between two word-vectors relative to a central or maximal point in a subspace should tell us something about the way that they interact with one another semantically: words with similarly lopsided co-occurrence profiles within a subspace will be skewed in the same direction, for instance, and so may be expected to share an affinity within the conceptual context being modeled. Relative distances from generic vectors and also from the origin might also be expected to predict semantic relationships between words. And finally, the characteristics of the space itself, potentially inherent in the generic vectors and their interrelationships outside any analysis of actual word-vectors, might tell us something about the underlying context of the generation of the space in the first place.

**Figure 2:**
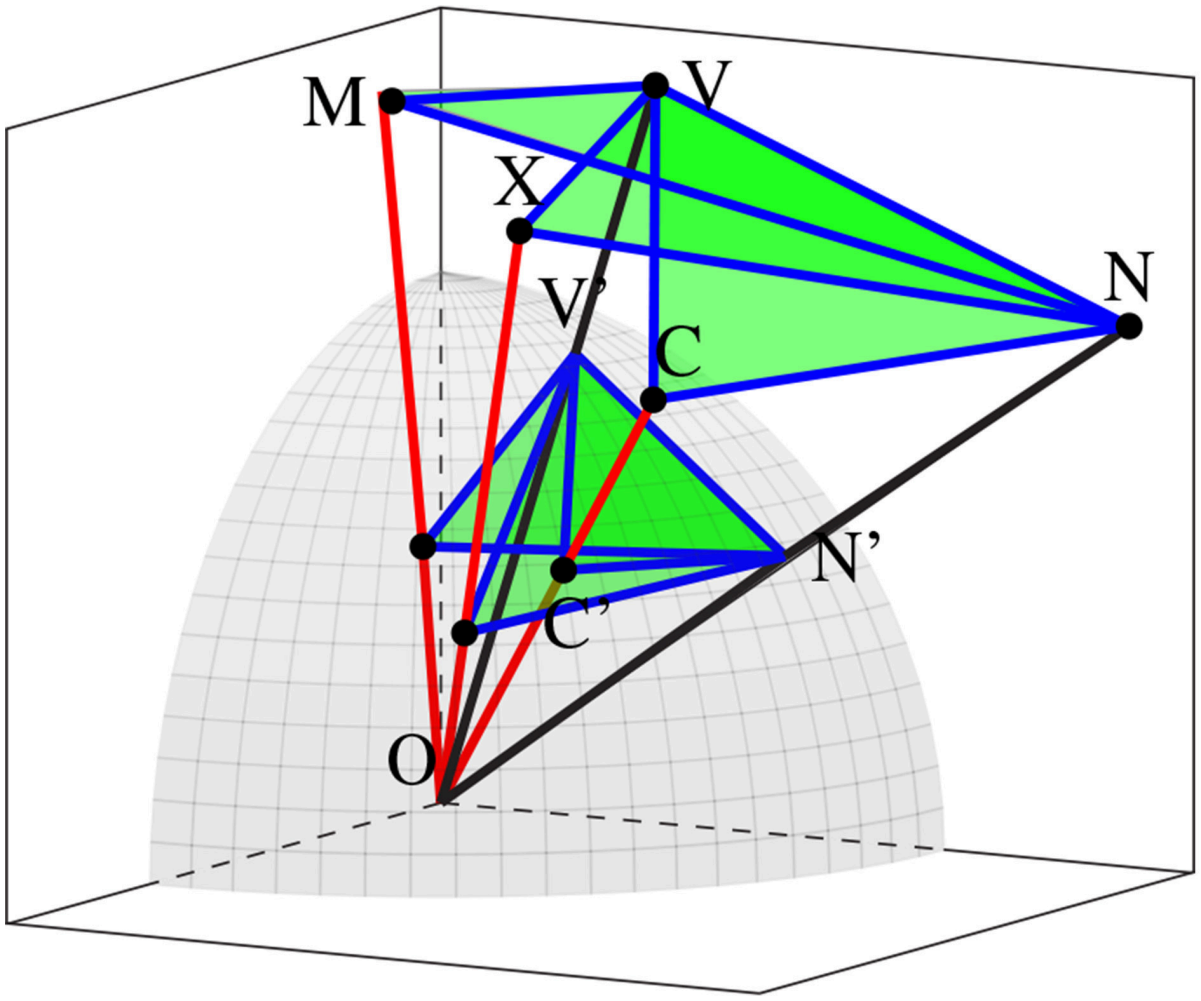
The geometry of a contextually projected subspace. *V* and *N* are verb and noun vectors, while *M*, *X*, and *C* are the mean, maximum, and central vectors. *V*′, *N*′, *M*′, *X*′, and *C*′ are their norms, where they intersect the unit sphere.

Figure 2 illustrates a subspace with all its characteristic features: the word vectors *V* and *N* which generate and then are subsequently projected into the subspace along with the mean, maximum, and central vectors, and then the various relationships which we propose to analyse in the context of metaphoricity. (*V* and *N* stand for *verb* and *noun*; as will be seen in section 4, the input to our space will be the components of potentially metaphoric verb-object phrases.) In addition to the aforementioned vectors, we also consider the normalized versions of each these vectors, which should provide us with a basis for considering the centrality of word-vectors. For instance, a verb-vector and noun-vector might have quite different lengths, and so could potentially form an obtuse angle with the mean-vector as a vertex (∠*VMN*), but they might both be to the same side of *M* in the space and so form an acute angle on a unit sphere (∠*V*′*M*′*N*′).

We define a total of 48 geometric features in any given subspace. These encompass distances, means of distances, ratios of distances, angles, areas of triangles defined by distances, and a number of these features taken at the surface of the hypersphere representing normalization of vectors. They are itemized in Table 1. Distances comprise the norms of vectors and the Euclidean distances between vectors, while means are the averages of some pairs of these distances. Ratios involve the fraction of the lower of a pair of distances over the higher, and are intended to provide a comparative measure of the relationship between vectors without presuming one as the numerator and the other as the denominator of a fraction. Fractions do take one vector norm or one mean of vector norms as an absolute denominator. Angles are taken both at the origin and at the vertices of generic vectors, and areas measure the triangles indicated by a subset of these angles.

**Table 1:** List of measures for geometric analysis of subspaces, with reference to Figure 2.

	**full vectors**	**normalized vectors**
*distances*	$\bar{V},\bar{N},\bar{VN},\bar{M},\bar{X},\bar{C}$	$\bar{V^{\prime }N^{\prime }}$
*means*	$\mu \left(\bar{VM},\bar{NM}\right),\mu \left(\bar{VX},\bar{NX}\right),\mu \left(\bar{VC},\bar{NC}\right)$	$\mu \left(\bar{V^{\prime }M^{\prime }},\bar{N^{\prime }M^{\prime }}\right),\mu \left(\bar{V^{\prime }X^{\prime }},\bar{N^{\prime }X^{\prime }}\right),$ $\mu \left(\bar{V^{\prime }C^{\prime }},\bar{N^{\prime }C^{\prime }}\right)$
*ratios*	$\left(\bar{VM}:\bar{NM}\right),\left(\bar{VX}:\bar{NX}\right),\left(\bar{VC}:\bar{NC}\right)$	$\left(\bar{V^{\prime }M^{\prime }}:\bar{N^{\prime }M^{\prime }}\right),\left(\bar{V^{\prime }X^{\prime }}:\bar{N^{\prime }X^{\prime }}\right),$
		$\left(\bar{V^{\prime }C^{\prime }}:\bar{N^{\prime }C^{\prime }}\right)$
*fractions*	$\bar{V}/\bar{N},\bar{VM}/\bar{NM},\bar{VX}/\bar{NX},\bar{VC}/\bar{NC},$ $\mu \left(\bar{V},\bar{N}\right)/\bar{M},\mu \left(\bar{V},\bar{N}\right)/\bar{X},\mu \left(\bar{V},\bar{N}\right)/\bar{C},$ $\bar{C}/\bar{M},\bar{C}/\bar{X},\bar{M}/\bar{X}$	$\bar{V^{\prime }M^{\prime }}/\bar{N^{\prime }M^{\prime }},\bar{V^{\prime }X^{\prime }}/\bar{N^{\prime }X^{\prime }},\bar{V^{\prime }C^{\prime }}/\bar{N^{\prime }C^{\prime }}$
*angles*	∠*VON*, ∠*VMN*, ∠*VXN*, ∠*VCN*, ∠*MOC*, ∠*MOX*, ∠*COX*	∠*V*′*M*′*N*′, ∠*V*′*X*′*N*′, ∠*V*′*C*′*N*′
*areas*	△*VMN*, △*VXM*, △*VCM*	△*V*′*M*′*N*′, △*V*′*X*′*M*′, △*V*′*C*′*M*′

Collectively, these measures describe all the components of the geometry of a contextualized distributional semantic subspace which we will explore for indications of metaphoric re-representation. In the experiments described in section 5, they will become the independent variables defining a set of models that will seek to learn to predict metaphoricity, meaningfulness, and familiarity in verb-object phrases. They will likewise serve as tools for interpreting the behavior of these models: the ability to trace these features back to co-occurrence phenomena will prove to be a useful mechanism for understanding the ways in which statistics derived from a large collection of text can be mapped to semantic phenomena associated with the contextualization inherent in conceptualisation.

### 3.5. Establishing a Baseline

In order to compare our dynamically contextual distributional semantic methodology, which has been specifically designed to capture the way that re-representation occurs in a cognitive and environmental context, with more standard distributional semantic techniques, we model our data using the word-vectors output by the widely reported word2vec methodology (Mikolov et al., 2013). This approach involves building a neural network which learns word-vectors by iteratively observing the ways that words co-occur in a corpus. The algorithm begins by randomly assigning each word in its vocabulary a word-vector in a normalized vector space, and then, each time a word is observed in a particular context, it adjusts the values of the corresponding word-vector slightly to pull it toward vectors corresponding to words observed in similar contexts.

The word2vec technique is different from our dynamically contextual approach in two important ways. First of all, it projects word-vectors into a normalized hypersphere of arbitrary dimensionality, meaning that the only measure for comparing two lexical semantic representations to one another is cosine similarity (which will correlate monotonically with Euclidean distance in a normalized space). This means that there is no mechanism for extracting the wider range of geometric features we use to examine the nuances of semantic phenomena, such as distance from origin, centrality, or relation to generic vectors.

Second, and perhaps even more importantly, because the word-vectors learned by a neural network are *abstract* in the sense that their dimensions are just arbitrary handles for making slight adjustments to relationships between vectors, there is no way to meaningfully select dimensions for the projections of lower dimensional subspaces corresponding to particular conceptual contexts. In fact, Levy and Goldberg (2014b) make a compelling case for considering this approach as being commensurate with the matrix factorization techniques for building semantic representations described by Deerwester et al. (1990), enhanced with a large number of modeling parameters.

We build a word2vec model based on the same corpus described in section 3.2, applying the *contextual bag-of-words* procedure outlined by Mikolov et al. (2013) to generate a 200 dimensional vector space based on observations within a 2 × 2 word co-occurrence window.^1^ This model will serve as a point of comparison with our own dynamically contextual distributional semantic methodology, offering up a singular space in which lexical semantic representations are simply compared in terms of their universal relationship to one another, without any mechanism for generating *ad hoc* relationships in a contextually informed way.

## 4. Human Metaphor Judgements

In this study, we seek to develop a computational model of the way that metaphor emerges in a particular conceptual context, as a linguistic artifact situationally endowed with an unfamiliar meaning. Our empirical objective will be to predict the extent to which multi-word phrases would be perceived as metaphoric. In order to generate data for this modeling objective, and also to understand the relationship between metaphor and other semantic categories, we introduce a dataset of verb-object compositions evaluated by human judges, and perform some preliminary analyses on correlations between the human judgements.

### 4.1. Materials

The materials are verb-noun word dyads, which were originally selected for an ERP study on metaphor comprehension in bilinguals (Jankowiak et al., 2017). Five normative studies were performed prior to the ERP experiment to confirm that the word pairs fell within the following three categories: novel metaphors (e.g., *to harvest courage*), conventional metaphors (e.g., *to gather courage*), and literal expressions (e.g., *to experience courage*). Based on the results of the normative studies, the final set of 228 English verb-noun word dyads (76 in each category) was selected for the purpose of the current study. The main results of the four normative studies performed prior to the EEG study will be reported here; for a more detailed discussion of the materials see Jankowiak et al. (2017). Mixed-design analyses of variance (ANOVAs) with utterance type as a within-subject factor and survey block as a between-subject factor were conducted. There was no significant main effect of block. Significance values for the pairwise comparisons were corrected for multiple comparisons using the Bonferroni correction. The Greenhouse-Geisser correction was applied whenever Mauchly's test revealed the violation of the assumption of sphericity, and in these cases, the original degrees of freedom are reported with the corrected *p*-value. Participation statistics are reported in Table 2.

**Table 2:** Demographic characteristics of participants of the four normative studies, including the number of participants (number of female participants) and mean age.

**Normative study type**	**Number of participants(female)**	**Mean age**
Cloze probability	140 (65)	23
Meaningfulness ratings	133 (61)	22
Familiarity ratings	101 (55)	23
Metaphoricity ratings	102 (59)	22

#### 4.1.1. Cloze Probability

To ensure that expectancy effects caused by participants anticipating the second word in a given word dyad would not impact the results of the EEG study, a cloze probability test was performed. Participants received the first word of a given word pair, and provided the second word, so that the two words would make a meaningful expression. If a given word pair was observed more than 3 times in the cloze probability test, the word dyad was excluded from the final set and replaced with a new one. This procedure was repeated until the mean cloze probability for word pairs in all four conditions did not exceed 8% [novel metaphoric, conventional metaphoric, and meaningless word pairs (*M* = 0, *SD* = 0); literal word pairs (*M* = 0.64, *SD* = 2.97)].

#### 4.1.2. Meaningfulness

Participants of this normative test rated how meaningful a given word pair was on a scale from 1 (totally meaningless) to 7 (totally meaningful). A main effect of utterance type was found, [*F*_(3, 387)_ = 1611.54, *p* < 0.001, ϵ = 0.799, η${}_{p}^{2}$ = 0.93]. Pairwise comparisons showed that literal word pairs were evaluated as more meaningful (*M* = 5.99, *SE* = 0.05) than conventional metaphors (*M* = 5.17, *SE* = 0.06) (*p* < 0.001), and conventional metaphors as more meaningful than novel metaphors (*M* = 4.09, *SE* = 0.08) (*p* < 0.001).

#### 4.1.3. Familiarity

Familiarity of each word pair was assessed in another normative study, in which participants decided how often they had encountered the presented word pairs on a scale from 1 (very rarely) to 7 (very frequently). A main effect of utterance type was found, [$F_{\left(2,296\right)}=470.97,p<0.001,\epsilon =0.801,\eta_{p}^{2}=0.83$]. Pairwise comparisons showed that novel metaphors (*M* = 2.15, *SE* = 0.07) were rated as less familiar than conventional metaphors (*M* = 2.97, *SE* = 0.08), (*p* < 0.001), with literal expressions being most familiar (*M* = 3.85, *SE* = 0.09), (*p* < 0.001). Furthermore, conventional metaphors were less familiar than literal word dyads, (*p* < 0.001). It should be noted that all word pairs were relatively unfamiliar, which is evident in the mean score for literal word pairs. They were evaluated as most familiar of all three categories, but did not obtain maximum familiarity values on the scale (below 4, while 6 and 7 represented highly familiar items). Familiarity was low in all three categories as we intentionally excluded highly probable combinations.

#### 4.1.4. Metaphoricity

In order to assess the metaphoricity of the word pairs, participants decided how metaphoric a given word dyad was on a scale from 1 (very literal) to 7 (very metaphoric). A main effect of utterance type was found, [$F_{\left(2,198\right)}=588.82,p<0.001,\epsilon =0.738,\eta_{p}^{2}=0.86$]. Pairwise comparisons showed that novel metaphors (*M* = 5.00, *SE* = 0.06) were rated as more metaphoric than conventional metaphors (*M* = 3.98, *SE* = 0.06, *p* < 0.001), and conventional metaphors were rated as more metaphoric than literal utterances (*M* = 2.74, *SE* = 0.07, *p* < 0.001).

### 4.2. Correlations in Human Judgements

In order to understand the way in which meaningfulness, familiarity, and metaphoricity interact in the judgements reported by humans, we model the correlations between each of these factors, as well as the propensity of each of these factors to identify the metaphoric class of a phrase (that is, whether it is literal, conventional, or novel). Results are reported in Table 3.

**Table 3:** Accuracy scores (for the class targets) and Pearson correlations (for the graded ratings) for semantic features of verb-noun pairs.

	**Class**	**Metaphoricity**	**Meaningfulness**	**Familiarity**
All others	0.737	0.686	0.734	0.714
Metaphoricity	0.715	-	–0.641	–0.613
Meaningfulness	0.579	–0.641	-	0.675
Familiarity	0.583	–0.613	0.675	-

The accuracy ratings for class are determined by performing a logistic regression taking the graduated human ratings for each semantic category as independent variables. Membership of each of the three candidate classes is determined through a one-vs.-rest scheme; the results in the class column of Table 3 are based on a leave-one-out cross-validation. In the case of *all others*, each of the three different semantic categories serve as the independent variables in a multi-variable logistic regression. Unsurprisingly, metaphoricity itself is most predictive of the metaphoric class of a phrase (*p* = 0.054 for the difference between metaphoricity and familiarity, based on a permutation test). The enhancement in accuracy by adding familiarity and meaningfulness to the model based only on metaphoricity is, on the other hand, not significant (*p* = 0.574).

**Figure 3:**
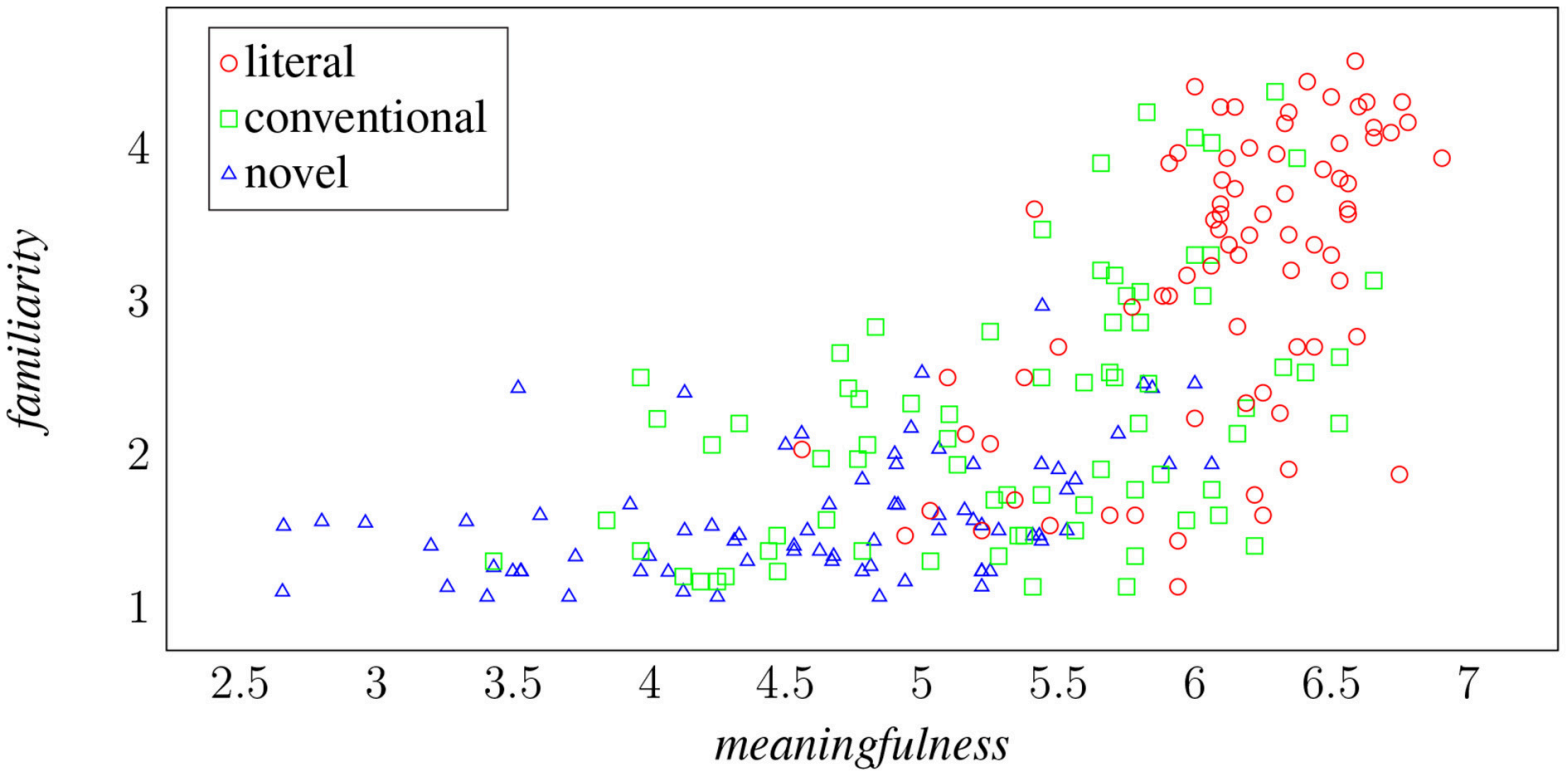
The three metaphoric classes as functions of meaningfulness and familiarity.

Figure 3 seeks to visualize the relationship between metaphoricity and the other two semantic phenomena measured here by projecting metaphoric classes of verb-object phrases in terms of meaningfulness and familiarity. The correlation between increases in familiarity and meaningfulness and the drift from literal phrases through conventional metaphors to novel metaphors is apparent, though there is also a good deal of overlap in the scores assigned to each category, with outliers from each class to found at all extents of the statistical cluster.

There are plenty of phrases that are considered meaningful but unfamiliar, and these phrases tend to be considered either literal or conventionally metaphoric, but there are very few phrases that are considered familiar and meaningless. It is tempting to therefore hypothesize that we might construe familiarity as, in itself, a product of meaning: there is an inherent relationship by which recognizing a semantic composition is contingent on recognizing its meaningfulness. More pertinently, we will claim that the process by which metaphor emerges from a cognitive re-representation of the world is evident in the way that humans judge these assessments of semantic categories to play out across these three classes of verb-object phrases. Those phrases that veer into the unfamiliar in particular are associated with the conceptual contortions implicit in novel metaphor.

## 5. Experimental Methodology

Building on the methodology for constructing a base space, projecting contextually informed subspaces from this base space, and extracting geometric features suitable for semantic analysis from these subspaces, we now turn to the project of applying this methodology to a model that captures the semantic assessments of humans. We apply the techniques outlined in section 3 to generate geometries associated with input in the form of verb-object phrases. We are effectively testing the degree to which human judgements of metaphor can be captured in statistical observations of word co-occurrences, and then exploring how these statistical tendencies can be contextually projected onto geometric features. Our modeling methodology will involve learning linear mappings between geometric features and human scores, as well as logistic regressions designed to predict metaphoric class.

In practice, this involves producing subspaces associated with each of the verb-object dyads in the dataset described in section 4. In these subspaces, the words composing the dyad are represented as vectors, and these vectors have a geometrical relationship to one another and to the subspace itself which can be represented as a feature vector (corresponding to the features described in Table 1). Our hypothesis is that these geometric features, which are designed to represent the semantics of the particular context associated with each input dyad, will map to ratings regarding the metaphoricity, meaningfulness, and familiarity of the dyad in question. This, returning to the theoretical background of section 2.3 and model of section 3.1, is intended to provide a computational mechanism that is conducive to modeling metaphor as a process of *ad hoc* concept construction within a particular communicative context.^2^

### 5.1. Modeling Metaphoric Re-representation From Geometries of Subspaces

We begin our experiments by building a base space of word-vectors based on a statistical analysis of Wikipedia, as described in section 3.2: this results in a matrix of information theoretical co-occurrence statistics. This matrix will serve as the basis for projections contextualized by particular verb-object compositions. In order to model the relationship between lexical semantic representations re-represented in potentially metaphoric contexts, we take each word pair in the dataset described in section 4.1 as input to each of the three subspace projection techniques described in section 3.3, working off the base space to generate 200 dimensional subspaces. We project the word-vectors associated with each input word into each subspace, and also compute the mean-vector, maximum-vector, and central-vector for each subspace. Based on these projections, we calculate the 48 geometric features listed in Table 1.

These features are then used as independent variables in least squares regressions targeting the human ratings for each of the three semantic categories assessed for each verb-object phrase: metaphoricity, meaningfulness, and familiarity.^3^ We pre-process the geometric measures by performing mean-zero, standard-deviation-one normalization across each feature. We similarly perform a logistic regression on the same normalized matrix of geometric features to learn to predict the metaphoric class (literal, conventional, or novel) of each dyad in our data. As with the model mapping from semantic ratings to classes described in section 4.2, we employ a one-vs.-rest scheme, so in effect we fit three different models, one for each class, and then classify a phrase based on the model for which that phrase scores highest.^4^ We once again employ a leave-one-out cross-validation technique.

The objective here is to evaluate the extent to which the geometric features of the subspaces we project collectively capture the contextual semantics of a particular dyad. By evaluating each dyad *d* on a regression of the 227 × 48 matrix of independent variables *D*′, defined such that *d*∉*D*′ (227 for all the dyads in our datasete except *d*, and 48 for the entire set of geometric features defined in Table 1), and then aggregating the average correlation scores across all dyads, we can get a general picture of the degree to which these features collectively correlate with human judgements.

### 5.2. Semantic Geometry

The full-featured approach described above offers a good overall sense of the way that statistical geometry maps to semantic features. There will, however, be a good deal of collinearity at play in the geometric features we have defined for our model. The angle between the verb and noun vectors, for instance (∠*VON* in Figure 2) would be expected to correlate somewhat with $\bar{VN}$, the Euclidean distance between the vectors. Likewise, the ratio of the smaller to the larger of distances between the word-vectors and the mean-vector $\bar{VM}:\bar{NM}$ will in many subspaces be identical to the fraction $\bar{VM}/\bar{NM}$.

To address this, we undertake a feature-by-feature analysis of our data. We isolate each of the 48 geometric features listed in Table 1 and calculate the Pearson correlation between the feature and the human ratings for each of the three semantic phenomena under consideration. This move provides the basis for an analysis of the way that specific aspects of the geometry of a contextualized subspace map to human judgements, which in turn allows us to tease out the specific correlations between co-occurrence statistics observed in a large-scale corpus and the re-representational processes associated with metaphor interpretation. In this sense, our subspace architecture becomes a geometric index mapping from the unstructred data available in a corpus to the dynamics of language in use.

### 5.3. Eliminating Collinearity

As mentioned above, there is inevitably collinearity between the geometric features we use to give analytical structure to our subspaces. Among other things, features corresponding to points of the normalized component of the geometry (so, *V*′, *C*′, *M*′, *X*′, and *C*′) will in many cases correlate with corresponding features associated with the non-normalized component of the geometry. In order to overcome this aspect of our geometric data, we apply a variance inflation factor to construct a reduced set of truly independent variables (O'Brien, 2007). This is effectively a statistic computed to iteratively build up a vector of adequately non-correlated geometric features by assessing the degree of covariance each additional feature would introduce to the aggregating set of features.

Our process begins by seeding an input matrix with the measures for each verb-object phrase for the top ranking geometric feature for a given semantic phenomena. We then move down the list of features, calculating the coefficient of determination *R*^2^ for a least squares linear regression between the established matrix and the measures associated with the next variable. We concatenate the next variable to our list of independent variables only if the following criterion is met:

$$\begin{array}{r} \frac{1}{1-R^{2}}<fac \end{array}$$

We set the model parameter *fac* at the quite stringent level of 2, and then select up to 5 out of the 48 features outlined in Table 1 as the independent variables for a linear regression trained on human ratings for three different semantic categories. We use this non-collinear set of features to run linear and logistic regressions to learn to predict semantic phenomena and metaphoric class respectively, applying once again leave-one-out cross-validations. This process results in a set of geometric features that we expect to be optimally informative in terms of correlations with human semantic judgements. This should offer us an opportunity to analyse in more detail the interactions between different features.

## 6. Results

Having established our experimental methodology, we apply the three different empirical stages outlined in section 5: a full-featured cross-evaluation of linear models mapping from the geometries of subspaces to human judgements of metaphoricity, meaingfulness, and familiarity; cross-evaluations of feature-by-feature linear models; and finally cross-evaluation of linear models constructed based on an iterative analysis designed to minimize collinearity between selected geometric features. Here we present results, with statistical significance calculated where appropriate, in terms of Fisher r-to-z transforms for rating correlations and permutation tests for classification f-scores.

### 6.1. Multi-Feature Correlations

Results for experiments involving linear models mapping all 48 geometric features of subspaces to graded human judgements of metaphoricity, meaningfulness, and familiarity are reported in the first three rows of Table 4. In the last row, labeled “class,” accuracy results for a logistic regression mapping from the full set of geometric features to human classifications of verb-object dyads as literal non-metaphors, conventional metaphors, or novel metaphors are reported. For these multi-feature correlations, we report results for all three subspace projection techniques: subspaces delineated by co-occurrence features independently selected based on the profile of each word in a dyad, and then subspaces selected based on the arithmetic and geometric means of co-occurrence features between the input words in a dyad.

**Table 4:** Pearson correlations for leave-one-out cross-validated linear regressions predicting semantic judgements based on geometric features extrapolated using three different subspace selection techniques, as well as with cosine similarity for the word2vec baseline.

	**INDY**	**MEAN**	**GEOM**	**w2v**	**Single-class baseline**
metaphoricity (correlation)	0.442	0.348	0.419	–0.288	-
meaningfulness (correlation)	0.430	0.380	0.290	0.215	-
familiarity (correlation)	0.452	0.283	0.391	0.224	-
class (accuracy)	0.447	0.447	0.442	0.458	0.333

*This is followed by accuracy for predicting the correct metaphoric class for each phrase*.

Interestingly, the features generated by the indy technique most closely reflect human judgements for all three semantic categories (though, even for the largest difference between the indy and mean techniques for familiarity, significance is marginal at *p* = 0.038 for a Fisher r-to-z transform). This is a bit less evident in terms of metaphoricity, where the geom technique achieves an appreciable correlation; nonetheless, it would appear that subspaces generated from the conjunction of dimensions independently salient to each of the two words involved in a phrase provide the most reliable geometric basis for predicting how humans will judge the phrase.

The results for predicting class are not significantly above the baseline accuracy score of 0.333 (indicated in the fifth column of Table 4), which would entail, for instance, predicting every phrase to be literal (*p* = 0.092 for the difference between this baseline and the indy output, based on a permutation test). Beyond that, the different subspace selection techniques are more or less in line with one another, suggesting that, more than for graduated human ratings of semantic phenomena, there is not much to choose between the different geometries generated here—at least when they are taken as a relatively high dimensional set of features entered into a regression model.

We compare these results with correlations and a logistic regression derived from the word2vec model described in section 3.5. As cosine similarity is the singular measure for judging the relationship between two words, we simply calculate the Pearson correlation between pairs of words in our input phrases and human ratings for the three graded semantic phenomena. We likewise perform a one-vs.-rest multi-class logistic regression to learn to predict the metaphoric class for each phrase. Results are reported in the fourth column of Table 4. The difference in metaphoricity scores between correlations with the indy technique and the word2vec baseline are not significant (*p* = 0.059 based on a Fisher r-to-z transform). Furthermore, word2vec is actually better at predicting the metaphoric class of a phrase than the model trained on all the geometric features of our model.

### 6.2. Single-Feature Correlations

There are a very large number of single-feature correlations to analyse: 48 separate ones, one for each component of the geometric feature map illustrated in Figure 2 and detailed in Table 1, multiplied by three different subspace projection techniques. We focus on the features extracted from subspaces generated using the indy technique, as the initial results from Table 4 suggest that these subspaces might be the most interesting from a semantic perspective. The top five features, in terms of the absolute value of correlation, are reported in Table 5, using the geometric nomenclature from Table 1 with reference to Figure 2.

**Table 5:** Top independent geometric features for three semantic phenomena as found in indy subspaces, ranked by absolute value of Pearson correlation.

**Metaphoricity**		**Meaningfulness**		**Familiarity**	
∠*VON*	–0.524	∠*VON*	0.451	∠*VMN*	0.431
$\bar{V^{\prime }N^{\prime }}$	0.519	$\bar{V^{\prime }N^{\prime }}$	–0.447	∠*VCN*	0.425
$\mu \left(\bar{V^{\prime }C^{\prime }};\bar{N^{\prime }C^{\prime }}\right)$	0.509	$\mu \left(\bar{V^{\prime }M^{\prime }};\bar{N^{\prime }M^{\prime }}\right)$	–0.437	$\mu \left(\bar{VC};\bar{NC}\right)$	–0.418
$\mu \left(\bar{V^{\prime }M^{\prime }};\bar{N^{\prime }M^{\prime }}\right)$	0.506	△*VXN*	–0.435	$\bar{V^{\prime }N^{\prime }}$	–0.407
△*VXN*	0.504	$\mu \left(\bar{V^{\prime }C^{\prime }};\bar{N^{\prime }C^{\prime }}\right)$	–0.433	∠*VON*	0.406

Not surprisingly, there is a degree of symmetry here: the results for metaphoricity and meaningfulness in particular come close to mirroring one another, with strongly positive correlations for one phenomena being strongly negative for the other, in line with the negative correlations between these phenomena as reported by humans in Table 3. The angle between the word-vectors, for instance (∠*VON*), correlates negatively with metaphoricity and positively with meaningfulness. This makes sense when we consider that a cosine relatively close to 1 between two vectors means that they are converging in a region of a subspace (regardless of their distance from the vector), and aligns with the strong results for cosine similarity achieved by our word2vec model, accentuated by the contextualization afforded by the indy contextualization technique.

What is perhaps surprising about these results is that there is such a clear, albeit inverse, correlation between the features that indicate metaphoricity and meaningfulness in these subspaces, while familiarity is associated with a slightly different geometric profile. This finding in regard to familiarity seems to stem from the non-normalized region of the subspace, suggesting that word-vectors that are not only oriented similarly but also have a similar relationship to the origin are more likely to be considered familiar. It would seem, then, that, in terms of the relationships between metaphoricity and meaningfulness, directions in a subspace are indicative of the semantic shift from the meaningful and known to metaphoric re-representation.

### 6.3. Optimized Correlations

Moving on from the single-feature analysis of each geometric feature of a particular type of subspace projection, we now turn to models built using multiple independent geometric features selected based on their independent performance constrained by a variance inflation factor, as described in section 5.3. To recapitulate, this involves adding one-by-one the top features as returned by the single-feature analysis reported above, so long as each additional feature does not exceed a value of 2 for the measure *fac* formulated in Equation 2, until at most five features are included in the optimized space of geometric features. Overall results for each subspace projection technique are reported in Table 6.

**Table 6:** Pearson correlations for leave-one-out cross-validated linear regressions predicting human judgements based on geometric features extrapolated using three different subspace selection techniques with up to 5 independent geometric features selected based on a variance inflation factor.

	**INDY**	**MEAN**	**GEOM**	**w2v**	**Single-class**
metaphoricity (correlation)	0.565	0.447	0.305	–0.288	-
meaningfulness (correlation)	0.492	0.428	0.255	0.215	-
familiarity (correlation)	0.464	0.383	0.318	0.224	-
class (accuracy)	0.531	0.465	0.412	0.458	0.333

Once again, the indy projection technique outperforms the other two techniques, as well as the word2vec baseline on all counts, including now accuracy of classification of verb-object dyads. There is a marked improvement for both the indy and mean techniques (*p* = 0.080 for the difference between the non-optimized and optimized indy metaphoricity predictions). The indy results are also improvements on the best scores for individual geometric features reported in Table 5, though the difference here is less pronounced. But on the whole, for these two techniques, there is clearly some advantage to discovering a set of non-collinear geometric features in order to understand how distributional statistics can be mapped to semantic judgements. Moreover, this refined version of our model outperforms the word2vec baseline in all regards, including prediction of metaphoric class, though the difference is not statistically significant (*p* = 0.247 for the difference between the indy technique and word2vec).

It is nonetheless interesting that a reduction in features motivated by observations about particular aspects of semantic geometry actually gives us a more productive model. As Guyon and Elisseeff (2003) point out, this is possibly an indicator of an underlying non-linearity between the geometric features of our subspaces and the human judgement of semantic properties. Given this, we may expect further improvement in results using for instance a neural modeling technique, but here our intentions are to explore the geometry of the subspaces in a straightforward and interpretable way, so we leave explorations of more computationally complex modeling for future study.

Table 7 focuses on the top features for each phenomenon as selected for the indy technique in particular. There are some telling trends here: where distance $\bar{V^{\prime }N^{\prime }}$ was independently predicative of all three semantic criteria in Table 5, this is hedged out by the even more predictive cosine measure ∠*VON* for metaphoricity and meaningfulness, because the correlation between $\bar{V^{\prime }N^{\prime }}$ and ∠*VON* is too high to satisfy *fac*. That these measures both correlate positively with meaningfulness is telling us that word-vectors detected to the same side of the middle of a subspace are more likely to form a meaningful composition and less likely to form a metaphorical one, but the presence of both of them in our analysis doesn't tell us much that the presence of one or the other wouldn't. A similar story can be told for the positive correlation of the angles at the vertices of both non-normalized mean and central vectors in the case of familiarity (∠*VMN* vs. ∠*VCN*). Again, it's not particularly surprising to see features like the mean distance between normalized word vectors and both normalized mean and central vectors achieving similar scores ($\mu \left(\bar{V^{\prime }M^{\prime }};\bar{N^{\prime }M^{\prime }}\right)$ vs. $\mu \left(\bar{V^{\prime }C^{\prime }};\bar{N^{\prime }C^{\prime }}\right)$).

**Table 7:** Top geometric features for three semantic phenomena as found in indy subspaces, ranked in the order that they are selected based on a variance inflation factor criterion, along with coefficients assigned in an all-in linear regression.

**Metaphoricity**		**Meaningfulness**		**Familiarity**	
∠*VON*	–0.297	∠*VON*	0.134	∠*VMN*	0.296
$\mu \left(\bar{VX};\bar{NX}\right)$	0.067	$\mu \left(\bar{VX};\bar{NX}\right)$	–0.111	$\mu \left(\bar{VX};\bar{NX}\right)$	–0.168
∠*V*′*X*′*N*′	–0.150	∠*V*′*X*′*N*′	0.157	△*VMN*	0.005
$\bar{VX}/\bar{NX}$	0.217	$\bar{VX}/\bar{NX}$	–0.249	$\bar{VC}/\bar{NC}$	0.184
$\bar{VX}:\bar{NX}$	0.162	$\bar{V^{\prime }C^{\prime }}:\bar{N^{\prime }C^{\prime }}$	–0.205	$\bar{VX}/\bar{NX}$	–0.050

To assess this final step in our modeling process in a little more detail, we consider the features themselves, along with the coefficients assigned to them in an all-in linear regression. These values are listed for the indy technique in Table 7. We once again note a strong negative correlation between the features that select for metaphoricity vs. the features that select for meaningfulness, with word-vectors that are found at wide angles (based on the ∠*VON* feature) and at relatively different distances from generic vectors (based on the $\bar{VX}/\bar{NX}$ and $\bar{VX}:\bar{NX}$ features) more likely to form a metaphoric composition.

Familiarity indicates a somewhat similar profile of features: like with meaningfulness, subspaces where the verb-vector and noun-vector are, on average, closer to the maximum extent of the space (*X*) tend to indicate a composition which humans will consider more familiar. The positive correlation of the fraction $\bar{VC}/\bar{NC}$ actually makes sense in relation to the (marginally) negative correlation with the fraction $\bar{VX}/\bar{NX}$, because we can expect to generally find the word-vectors that select these subspaces in the region between the central-vector *C* and the maximum-vector *X*. So it would seem that, as with meaningfulness, as the verb-vector grows relatively closer to *X* compared to the noun-vector, phrases are more likely to be familiar to humans.

## 7. Discussion

Having established the results of our dynamically contextual methodology's ability to model human judgements of metaphoricity, meaningfulness, and familiarity, we turn to an analysis of the components of our experimental set-up. In addition to an overall assessment of the methodology and a consideration of performance of certain parameter settings and particular geometric features, we would like to emphasize the way that the combination of subspace projection and linear feature mapping works to provide the framework for a more nuanced consideration of the relationship between corpus analysis and the cognitive and linguistic components of semantic phenomena. Our overall claim is that the context-specific and geometrically nuanced approach we have endorsed here shows promise as a way for using computational modeling to explore language as a fundamental component of human behavior.

### 7.1. Model Parameters

One of the findings that emerges from the results presented in section 6 is an opportunity to compare different modeling parameters, and to consider the relationship between these components of our methodology and metaphoric re-representation. The modeling feature that is of most interest here is the difference between the indy, mean, and geom subspace projection techniques, and the primary thing to note is the superior performance of the indy technique in modeling human considerations of all three semantic phenomena investigated here: metaphoricity, meaningfulness, and familiarity.

We begin by recalling that, as mentioned in section 3.3, the mean and geom techniques are really two different ways of computing average values of co-occurrence features potentially shared between different input words, while the indy technique produces a subspace that is a mixture of co-occurrence features that are independently salient to one word or the other—or possibly, but not necessarily, both. In fact, what we might be seeing in the strong correlations between geometric features of the indy subspaces and human judgements is, in part, the identification of instances where the co-occurrence profiles of input words tend to converge of diverge. This claim is supported by the strong negative correlation between metaphoricity and cosine (∠*VON*) in Table 7, along with the positive correlation with the mean distance of the vectors from the maximal point *X*, and the opposite set of correlations for the same features observed for meaningfulness. As the set of independently selected co-occurrence features evidence less overlap for the two components of the verb-object input dyad, the angle of the contextually projected word-vectors corresponding to these inputs drift apart in the subspace, and the regions of the projection become less correspondent with one another.

Additionally, the geom methodology actually realizes lower Pearson correlations for non-collinear combinations of geometric features than it does for the full set of geometric features. The definitive aspects of this technique are that it only selects co-occurrence dimensions with non-zero values for both input words, and that it furthermore tends to favor dimensions where the value is pretty high for both input words rather than very high for one and not so high for the other [the geometric mean of (5,5) is 5, but for (9,1) it is only 3]. These subspaces therefore should already exhibit a good degree of information about both word-vectors of a verb-object phrase, so there is perhaps less to be discovered in measures such as angular divergences relative to generic vectors near the center of a subspace. On the other hand, the requirement for mutually non-zero co-occurrence dimensions means that co-occurrences with relatively common words will eventually have to be selected, and so we might find information about co-occurrence features that are not in any sense conceptually salient, but instead just happen to come up quite often in our corpus. We could hypothesize that a larger co-occurrence window would yield stronger predictions for these subspaces, since there would be more observations of co-occurrences in the corpus for any given word-vector. We leave further experimentation along these lines for future work.

### 7.2. Using Geometry to Interpret Semantics

The analysis offered above of the strong performance of the indy subspace selection technique is indicative of the general way in which we would like to suggest that statistical geometries can be mapped to semantic phenomena. The combination of interpretable projections and nuanced analysis of the way that input word-vectors tend to move around relative to contexts associated with a set of graded semantic measures turns the list of geometric features enumerated in Table 1 into a set of semantic indices, providing traction for using modeling techniques that move from statistics about word co-occurrences to commitments about the way that humans use metaphor. In this way, geometric analysis maps to cognitive phenomena, elevating the model from something that merely learns to predict correlations to something that captures the way concepts are manipulated and indeed generated in response to an unfolding environment.

The divergence between the relatively congruent, albeit converse, features that model metaphoricity and meaningfulness as compared to the features that model famliarity offers a case in point. There is a close semantic relationship between metaphor and meaning: we might argue that a metaphor involves shifting a concept to suit a situation, and new meaning is produced as a result of this shifting. Familiarity, on the other hand, is an epistemological phenomenon with a frequentist connotation, and so is not expected to map neatly to this relationship between metaphor and meaning. This disconnect seems to play out in the interpretable geometry of context specific subspaces projected by our model. In the geometric features that provide traction to our model, the non-linear tension between familiarity and meaningfulness as reported by humans and illustrated in Figure 3 is teased out in terms of the distinct set of geometric features associated with familiarity. In particular, in Tables 5, 7, we see that familiarity has a relationship with the mean point *M* in contextual subspaces, suggesting that the relationship between projected word-vectors relative to the typical non-zero characteristics of a projection tell us something about how readily accepted a composition will be to humans.

### 7.3. The Dynamic Geometry of Representation

In order to examine more closely the nature of re-representation by way of contextualized projections of statistical geometry, we look at two case studies. Each case involves one noun applied to three different verb-object phrases, one judged to be literal, one conventionally metaphoric, and one a novel metaphor, as outlined in section 4.1. Our objective is to offer a qualitative, visually grounded analysis of the way that the typical geometry of projections shifts as we move across the spectrum of metaphoricity.

**Figure 4:**
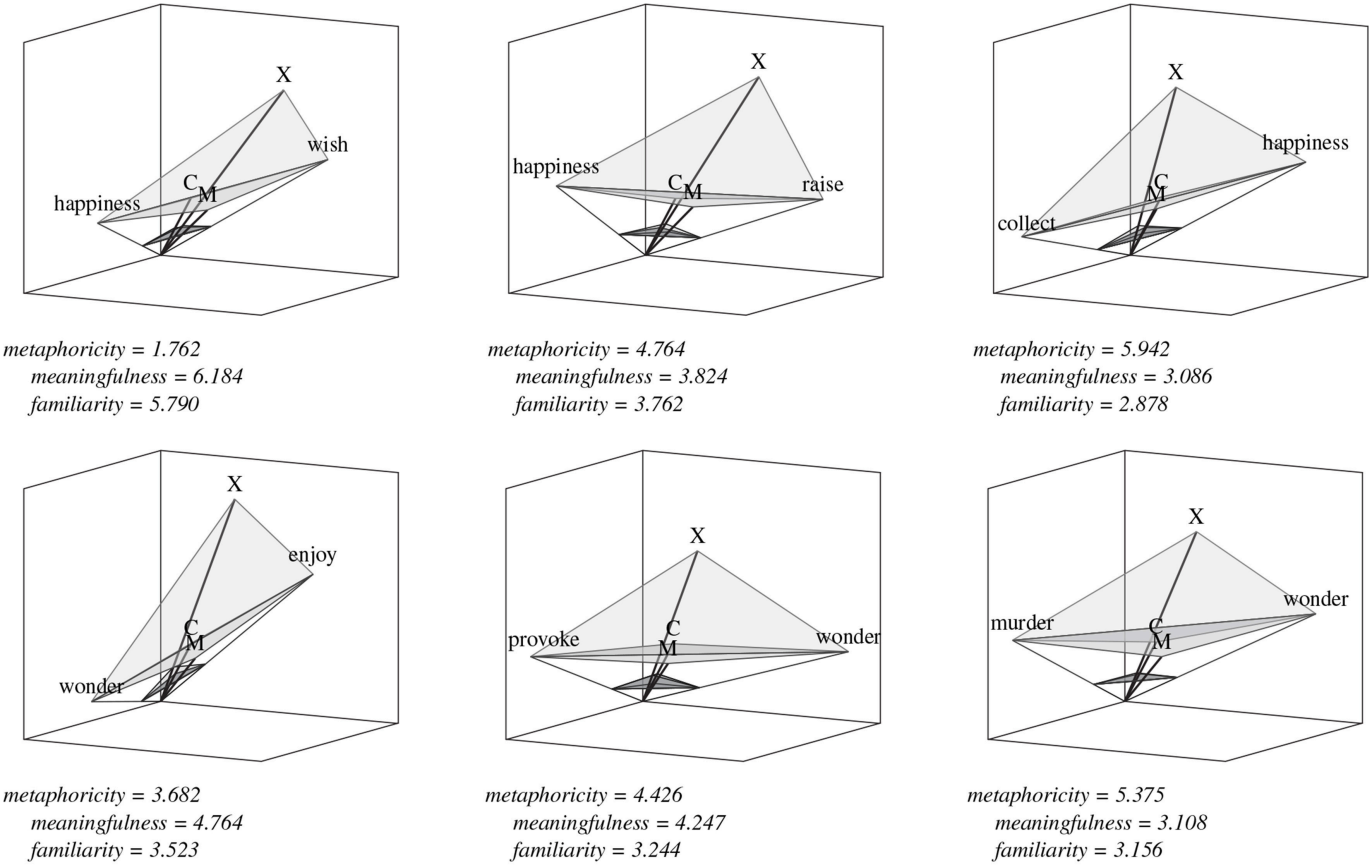
Subspaces, including word-vectors and generic features, for two different nouns composed with three verbs each, ranging from literal on the left to novel metaphor on the right. These three-dimensional projections have been derived through a regression designed to preserve the norms of all vectors, the distances between the word vectors, and the distances between each word-vector and all the generic vectors. The ratings assigned by our model are indicated below each plot.

Our two examples are presented in Figure 4, where the word-vectors and generic vectors as projected into 200 dimensional subspaces using the indy subspace selection technique are further projected into perspectives on three-dimensional renderings. These instances have been selected because the ratings output for metaphoricity by our model follow a regular progression as we move from literal to conventional to novel compositions. The first example involves the phrases *wish happiness, raise happiness*, and *collect happiness*; the second example involves the phrases *enjoy wonder, provoke wonder*, and *murder wonder*. With each noun, metaphoricity as rated by our model progressively increases with each successive composition, and meaningfulness and familiarity conversely decrease.

Along with this progression, we observe a gradual expansion of the complexes of vectors as we move from the literal to the overtly metaphoric. This is in line with the widening of the angle ∠*VON*, as statistically observed in Table 6. We also note an extension of the maximal-vector *X* away from the other points of interest in a subspace, a characteristic predicted by the increase of the mean distance between the word vectors and the maximal-vector $\mu \left(\bar{VX}:\bar{NX}\right)$. In terms of the spreading of the angle ∠*VMC* characteristic of decreasing familiarity, this is harder to perceive in this visualization, but there is a detectable flattening of the already wide vertices at both *M* and *C* by the time we get to *collect happiness* in particular.

In the end, it is difficult to make any very precise observations about these figures. They are necessarily lossy projections from much higher dimensional spaces, and the tricks of perspective when rendering three dimensions onto a plane also means that information about angular relationships even in these low-dimensional projections is easily lost. The purpose of these last illustrations is not so much to provide a tool for rigorous quantitative analysis, which has been provided above, as to show in a more general and qualitative sense that there is a spatial quality to the way that metaphor emerges as we edge away from the familiar and the meaningful. We argue that this quality corresponds to the re-representation inherent in constructing novel ways of talking about situations in the world.

Perhaps the appropriate way to think about metaphoric re-representation is in terms of a discovery of unfamiliar meaning in a particular context. So, while both humans and our computational model tend to identify a negative correlation between meaningfulness and metaphoricity, we could imagine how phrases like *collect happiness* and *murder wonder* could gain potent semantics in the right situation. Our computational model, underwritten by concrete and quantifiable observations of the way that words tend to be used, is designed to extrapolate a more general geometric way of capturing the process by which contextualization leads to the *ad hoc* construction of new representations with very specific communicative potentialities. Without wanting to make too strong a claim about what we can expect from computational models, we suggest that this geometric mode of representing metaphor in terms of statistical information about large-scale co-occurrence tendencies hints at a move toward a computational methodology for capturing some of the non-propositional and phenomenological components of figurative language (Davidson, 1978; Reimer, 2001; Carston, 2010b).

## 8. Conclusion

We argue here that dynamically projecting context-specific conceptual subspaces into new representations captures the mapping process that is necessary for conceptually resolving the semantics of non-literal language. We hypothesized that the geometry defining these subspaces (which reflects lexical co-occurrence relationships in a large-scale textual corpus) can be thought of as a quantification of the process of re-representation. This allows us to examine how the conceptual re-mappings underlying metaphoric language perception are related to underlying mathematically-tractable lexical semantic representations. By examining features of contextualized subspaces, our novel methodology can be used to assess the way that the overall geometric quality of a representation in our model maps to metaphoric shifts in meaning. We believe that this aspect of our approach may point the way toward the computational modeling of some of the more elusive theoretical properties of figurative language as a cognitive mechanism for moving away from propositional content.

Our methodology has been designed to accommodate pragmatic accounts of metaphor, by which figurative compositions involve the construction of an *ad hoc* conceptual space: the subspaces projected by our dynamically contextual model correspond to these extemporaneously projected semantic relationships. This facility is not intended to come at the expense of other accounts of metaphor; rather, we have been motivated by exploring ways that a theoretical stance that has typically proved challenging for computational semantic modeling can be addressed within the broader paradigm of distriubtional semantics.

With this in mind, we can imagine ways that future development of our methodology might lend itself to practical applications in neurolinguistic and clinical contexts. For instance, experimental evidence indicates major deficits in metaphoric language in conditions such as schizophrenia (Bambini et al., 2016): our methodology could provide a quantitative tool for introducing this pragmatic component to predict clinical diagnosis, as proposed for other aspects of language (Foltz et al., 2016). More generally, our approach can be counted as a contribution to a growing body of literature that seeks to use data-drive techniques to make links between neurolinguistic studies and some of the more complex aspects of language in use (Jacobs and Kinder, 2017), epitomized by the contextually situated re-representation at play in the use of metaphor.

## Author Contributions

SM: lead author, primary architect of computational model; KA: contributed to overall article, particularly psycholinguistic research; KR: contributed writing in psycholinguistic section of paper, also responsible for the dataset we used; MP: contributed to overall article, particular sections describing distributional semantic methods; GW: contributed to overall article, particularly regarding modeling commitments and results.

### Conflict of Interest Statement

The authors declare that the research was conducted in the absence of any commercial or financial relationships that could be construed as a potential conflict of interest.

## Footnotes

^1^This is implemented using the Gensim module for Python.

^2^Scripts for building dynamically contextual distributional semantic models, as well as for using these models to project context-specific subspaces and use these subspaces to model human metaphor judgements, are available at https://github.com/masteradamo/metaphor-geometry. The data on human metaphor judegements is available at https://figshare.com/articles/To_Electrify_Bilingualism_Electrophysiological_Insights_into_Bilingual_Metaphor_Comprehension/4593310/1; this data is described in detail by Jankowiak et al. (2017).

^3^This is implemented using the sklearn LinearRegression module for Python.

^4^This is implements using the sklearn LogisticRegression module for Python.
